# Endometrial epithelial cells-derived exosomes deliver microRNA-30c to block the BCL9/Wnt/CD44 signaling and inhibit cell invasion and migration in ovarian endometriosis

**DOI:** 10.1038/s41420-022-00941-6

**Published:** 2022-04-02

**Authors:** Mengmeng Zhang, Xi Wang, Xiaomeng Xia, Xiaoling Fang, Tingting Zhang, Fengying Huang

**Affiliations:** 1 Department of Obstetrics and Gynecology, The Second Xiangya Hospital, Central South University, Changsha, Hunan 410000 P.R. China; 2 Department of Obstetrics and Gynecology, Women’s Hospital, School of Medicine, Zhejiang University, Hangzhou, Zhejiang 310006 P.R. China

**Keywords:** Cell biology, Diseases

## Abstract

Endometriosis (EMs) is a benign gynecological disorder showing some tumor-like migratory and invasive phenotypes. This study intended to investigate the role of microRNA-30c (miR-30c) in EMs, which is involved with B-cell lymphoma 9 (BCL9), an activator of the Wnt/β-catenin signaling pathway. EMs specimens were clinically collected for determination of miR-30c and BCL9 expression. Exosomes were isolated from endometrial epithelial cells (EECs), and the uptake of exosomes by ectopic EECs (ecto-EECs) was characterized using fluorescence staining and confocal microscopy. The binding of miR-30c to BCL9 was validated by dual-luciferase reporter assay. Artificial modulation (up- and down-regulation) of the miR-30c/BCL9/Wnt/CD44 regulatory cascade was performed to evaluate its effect on ecto-EEC invasion and migration, as detected by Transwell and wound healing assays. A mouse model of EMs was further established for in vivo substantiation. Reduced miR-30c expression and elevated BCL9 expression was revealed in EMs ectopic tissues and ecto-EECs. Normal EECs-derived exosomes delivered miR-30c to ecto-EECs to suppress their invasive and migratory potentials. Then, miR-30c was observed to inhibit biological behaviors of ecto-EECs by targeting BCL9, and the miR-30c-induced inhibitory effect was reversed by BCL9 overexpression. Further, miR-30c diminished the invasion and migration of ecto-EECs by blocking the BCL9/Wnt/CD44 axis. Moreover, miR-30c-loaded exosomes attenuated the metastasis of ecto-EEC ectopic nodules. miR-30c delivered by EECs-derived exosomes repressed BCL9 expression to block the Wnt/β-catenin signaling pathway, thus attenuating the tumor-like behaviors of ecto-EECs in EMs.

## Introduction

Endometriosis (EMs) represents a chronic, inflammatory gynecological condition characterized by the presence of the uterine lining outside the uterine cavity [1]. EMs can be anatomically divided into three sub-groups: superficial peritoneal endometriosis, deeply infiltrating endometriosis, and ovarian endometriosis (O-EMs) [2]. Notably, O-EMs is accompanied by an elevated risk of ovarian cancer [3], impaired ovarian reserve, and sterility [4], as well as the failure of in vitro fertilization [5]. EMs, as a benign disease, present similar biological characteristics with cancers like invasive and migratory properties, development of local and distant foci, and resistance to apoptosis [6, 7]. Unfortunately, the etiology of EMs remains elusive, and current therapies for this disease are ineffective in the long term for many patients, accompanied by a high risk of recurrence [8]. Hence, investigations on novel therapeutic regimens for EMs are of urgent need.

Extracellular vesicles (EVs), especially exosomes, have been well-established to deliver microRNAs (miRNAs), proteins, and lipids, and the cargos have been assumed to work cooperatively to modulate tumor microenvironment [9]. Intriguingly, evidence exists suggesting that exosomes secreted by ectopic endometrial stromal cells could restrict the fibrosis in EMs by delivering a typical miRNA, miR-214 [10, 11]. Further, another miRNA, miR-30c, has been highlighted for notably downregulated expression in ectopic and eutopic endometriosis tissues [12]. Moreover, miR-30c-5p loaded by urinary exosomes has been recognized as a biomarker for clear cell renal cell carcinoma [13], and exosomal miR-30c has also been involved in the diagnosis of pancreatic cancer [14]. In this sense, this study focused on the potential role of exosomal miR-30c in EMs.

Furthermore, it has been previously suggested that miR-30c may negatively regulate B-cell lymphoma 9 (BCL9) and affect the Wnt/β-catenin signaling pathway, thereby attenuating the biological phenotypes of multiple myeloma cells [15]. BCL9 is a well-recognized oncogene that acts as a transcriptional co-activator of the Wnt/β-catenin pathway and has been implicated in a variety of cancers [16, 17]. Additionally, a prior study has pointed out that BCL9 could enhance β-catenin-mediated transcriptional activity, and that BCL9 knockdown reduced tumor burden, metastasis, and angiogenesis *via* inhibition on the expression of c-Myc, cyclin D1, cluster of differentiation 44 (CD44), and vascular endothelial growth factor [18]. Moreover, CD44 functions as a well-described target gene of the Wnt/β-catenin signaling pathway [19].

Based on the aforementioned evidence, we hypothesized in the present study that exosomal miR-30c may negatively regulate BCL9 and affect the activity of the Wnt/CD44 signaling pathway, thereby restricting the progression of EMs.

## Results

### MiR-30c is poorly expressed and BCL9 is highly expressed in O-EMs

**Fig. 1:**
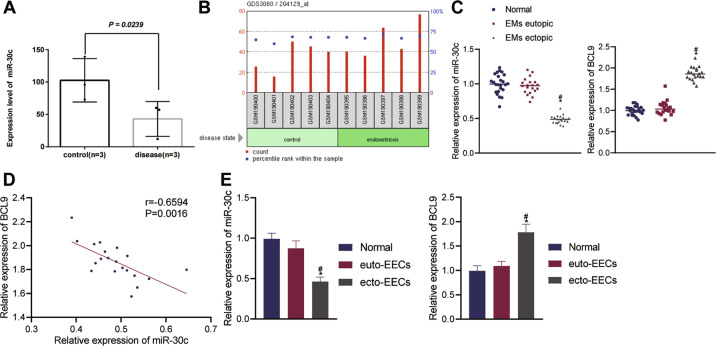
miR-30c expression was downregulated and BCL9 expression was upregulated in EMs tissues and cells. **A** High-throughput transcriptome sequencing of miR-30c in eutopic and ectopic endometrial tissues of O-EMs patients and normal endometrial tissues (control); **B** Expression of BCL9 in EMs eutopic and ectopic tissues and normal endometrial tissues (control) in GSE7846 EMs-related microarray; **C** Expression levels of miR-30c and BCL9, determined by qRT-PCR, in 24 cases of EMs eutopic and ectopic tissues and 20 cases of normal endometrial tissues (Normal); **D** Pearson correlation analysis of the correlation between miR-30c and BCL9 expression in EMs ectopic tissues; **E** Expression levels of miR-30c and BCL9, determined by qRT-PCR, in ecto-EECs, euto-EECs, and normal EECs. Measurement data were characterized as mean ± SD. The comparison among data of multiple groups was performed with one-way ANOVA with Tukey’s post hoc test. Pearson correlation was used to analyze the correlation between miR-30c and BCL9 expression. *p < 0.05 versus the Normal group, #*p* < 0.05 versus the EMs eutopic tissue or the euto-EECs.

Through the high-throughput transcriptome sequencing of the eutopic and ectopic endometrial tissues of O-EMs patients, we identified the downregulated expression of miR-30c in ectopic endometrial tissues relative to normal endometrial tissues (Fig. 1A). Differential analysis of the GSE7846 microarray indicated that BCL9 was upregulated in the ectopic endometrial tissues (Fig. 1B).

Subsequently, we determined the expression of miR-30c and BCL9 in 24 cases of O-EMs eutopic and ectopic endometrial tissues and 20 cases of normal endometrial tissue. Results of quantitative reverse transcription PCR (qRT-PCR) confirmed the underexpression of miR-30c and the overexpression of BCL9 in O-EMs ectopic tissues (Fig. 1C). Pearson correlation analysis of the expression of miR-30c and BCL9 in O-EMs ectopic tissue further indicated that miR-30c expression was negatively correlated with BCL9 expression (Fig. 1D). Moreover, we measured the expression of miR-30c and BCL9 in ecto-EECs, euto-EECs, and normal EECs, and the results were consistent with those in clinical tissues (Fig. 1E). Underexpression of miR-30c and overexpression of BCL9 occurred in ecto-EECs.

Collectively, our results revealed the downregulated expression of miR-30c and upregulated expression of BCL9 in O-EMs, indicating their involvement in the pathogenesis of O-EMs.

### EECs-derived exosomes repressed the invasion and migration of ecto-EECs by delivering miR-30c

**Fig. 2:**
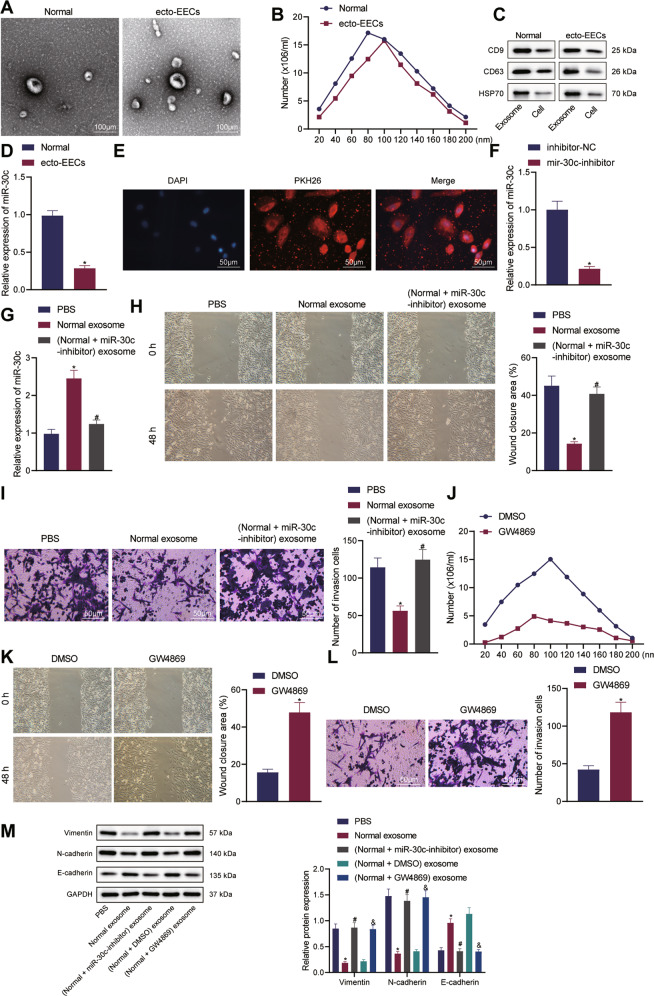
EECs-derived exosomes deliver miR-30c to repress the invasion and migration of ecto-EECs. **A** TEM observation of exosomes derived from ecto-EECs and normal EECs; **B** Nanoparticle tracking analysis of the size and distribution of exosomes derived from ecto-EECs and normal EECs; **C** Expression levels of CD9, CD63, and HSP70 in isolated exosomes and cell extracts, determined by Western blot assay; **D** The expression of miR-30c in exosomes derived from ecto-EECs and normal EECs, determined by qRT-PCR; **E** Immunofluorescence detection of ecto-EECs cocultured with PKH26-stained exosomes derived from normal EECs (Red indicates exosomes and blue indicates DAPI-stained nuclei); **F** The expression of miR-30c in exosomes derived from miR-30c-inhibiting normal EECs, determined by qRT-PCR; **G** The expression level of miR-30c in ecto-EECs following coculture with exosomes from miR-30c-inhibiting normal EECs, determined by qRT-PCR; **H** Wound healing assay to detect cell migration in ecto-EECs following coculture with exosomes from miR-30c-inhibiting EECs, reflected by quantification of the wound healing rate; **I** Transwell assay to detect cell invasion in ecto-EECs after coculture with exosomes from miR-30c-inhibiting EECs, with the number of invasion cells counted; **J** Nanoparticle tracking analysis of the size distribution and number of exosomes derived from GW4869-treated normal EECs (GW4869, an exosome release inhibitor); **K** Wound healing assay to detect cell migration in ecto-EECs following coculture with exosomes from GW4869-treated normal EECs, reflected by quantification of the wound healing rate; **L** Transwell assay to detect cell invasion in ecto-EECs after coculture with exosomes from GW4869-treated normal EECs, with the number of invasion cells counted; **M** Expression levels of EMT-related proteins in ecto-EECs cocultured with exosomes from EECs of different groups, determined by Western blot. Cellular experiments were repeated three times. Measurement data were characterized as mean ± SD. The comparison between data of two groups was performed by independent sample *t*-test, and that among multiple groups was performed with one-way ANOVA with Tukey’s post hoc test. *p < 0.05 versus the Normal group, inhibitor-NC group, PBS group, or DMSO group; #*p* < 0.05 versus the Normal exosome group; &*p* < 0.05 versus the (Normal + DMSO) exosome group.

To examine whether miR-30c was delivered to EECs through exosomes to participate in the progression of O-EMs, we isolated exosomes from the culture supernatant of ecto-EECs and normal EECs. Transmission electron microscope (TEM) observations validated that the exosomes secreted by the two kinds of EECs were double-membrane vesicles with a size of 50–100 nm (Fig. 2A). Nanoparticle tracking analysis further confirmed the size of exosomes to be around 80 nm (Fig. 2B). Western blot assay was then performed to determine the expression of exosome markers (CD9, CD63, and HSP70) in exosomes and cell extracts, and the results displayed that CD9, CD63, and HSP70 were highly expressed in the extracted exosomes, while HSP70 was mainly distributed in cell extracts, demonstrating successful isolation of exosomes (Fig. 2C). In addition, qRT-PCR results displayed lower expression of miR-30c in exosomes derived from ecto-EECs, relative to that from normal EECs (Fig. 2D).

To further explore whether miR-30c was delivered by exosomes, we stained the normal EECs-derived exosomes with PKH26 and cocultured them with ecto-EECs for 48 h. As a result, a strong red signal was then observed in ecto-EECs (Fig. 2E). Exosomes derived from miR-30c-inhibiting normal EECs, as shown by qRT-PCR, presented with a decreased expression of miR-30c (Fig. 2F). Moreover, ecto-EECs cocultured with exosomes isolated from normal EECs exhibited upregulated expression of miR-30c, while those cocultured with exosomes from miR-30c-inhibiting EECs showed no obvious changes in miR-30c expression (Fig. 2G). These results unraveled that miR-30c was loaded by exosomes and shuttled from normal EECs to ecto-EECs.

Next, we explored the effect of miR-30c delivered by exosomes on the biological behaviors of ecto-EECs. Results of the Transwell and wound healing assays showed that the invasion and migration of ecto-EECs were obviously suppressed in response to the coculture with miR-30c-loaded exosomes, and that additional inhibition of miR-30c negated the suppression (Fig. 2H, I).

Furthermore, we treated normal EECs with the exosome secretion inhibitor GW4869 and found through nanoparticle tracking analysis that the number of exosomes was reduced following GW4869 exposure (Fig. 2J). It was also observed that GW4869 treatment diminished the suppressing effects of normal EECs-derived exosomes on ecto-EEC migration and invasion (Fig. 2K, L). Consistently, the protein expression of epithelial-mesenchymal transition (EMT)-related Vimentin and N-cadherin was decreased and that of EMT-related E-cadherin was increased in ecto-EECs after coculture with normal EECs-derived exosomes, and such effects of EECs-derived exosomes were reversed by either miR-30c knockdown or GW4869 treatment (Fig. 2M).

Taken together, our results indicated that exosomes derived from normal EECs suppressed the invasive and migratory potentials of ecto-EECs by delivering miR-30c.

### MiR-30c diminishes the invasion and migration of ecto-EECs through targeting BCL9

**Fig. 3:**
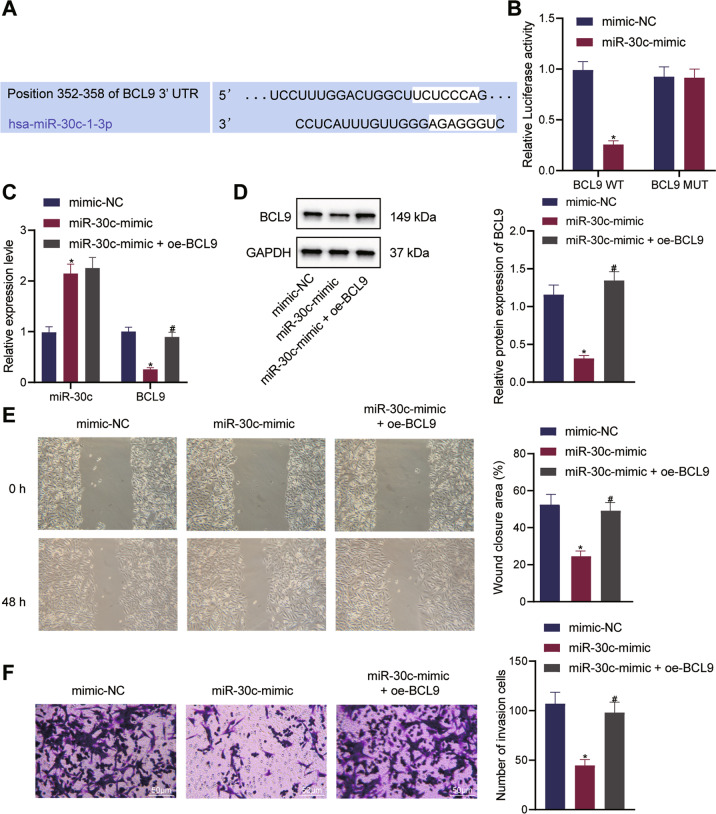
miR-30c targeted and inversely regulated BCL9, thus repressing ecto-EEC migratory and invasive phenotypes. **A** Online prediction of the binding site of BCL9 and miR-30c by TargetScan; **B** Dual-luciferase reporter gene assay to verify the binding of miR-30c to BCL9; **C** qRT-PCR measurement of the expression of miR-30c and BCL9 in ecto-EECs in response to the overexpression of miR-30c alone or in combination with BCL9; **D** Western blot measurement of the protein expression of BCL9 in ecto-EECs in response to the overexpression of miR-30c alone or in combination with BCL9; **E** Wound healing assay to detect the migration of ecto-EECs in response to the overexpression of miR-30c alone or in combination with BCL9; **F** Transwell assay to detect the invasion of ecto-EECs in response to the overexpression of miR-30c alone or in combination with BCL9. Cellular experiments were repeated three times. Measurement data were characterized as mean ± SD. The comparison between data of two groups was performed by independent sample *t*-test and that among multiple groups was performed with one-way ANOVA with Tukey’s post hoc test. *p < 0.05 versus the mimic-NC group, #*p* < 0.05 versus the miR-30c-mimic group.

Following the aforementioned findings, we then explored the interaction between miR-30c and BCL9. First, miR-30c binding sites on BCL9 was predicted by the TargetScan online tool (Fig. 3A). The direct binding of miR-30c to BCL9 was then validated by dual-luciferase reporter gene assay (Fig. 3B). Further, we overexpressed miR-30c alone or in combination with BCL9 in ecto-EECs. qRT-PCR and Western blot measurements confirmed that miR-30c expression was increased and BCL9 expression was reduced in response to miR-30c-mimic treatment alone and that simultaneous overexpression of BCL9 led to upregulated BCL9 expression and no obvious change in miR-30c expression (Fig. 3C, D). These results demonstrated that miR-30c targeted BCL9 and inhibited its expression.

Next, we performed functional assays to detect the role of the miR-30c/BCL9 regulatory axis in the biological behaviors of ecto-EECs. As shown by Transwell and wound healing assays, cell invasion and migration in ecto-EECs were attenuated in the presence of miR-30c overexpression, which was then abrogated when BCL9 was also overexpressed (Fig. 3E, F).

In summary, these results revealed that miR-30c restricted the invasion and migration of ecto-EECs by inversely regulating BCL9 and that BCL9 overexpression reversed the effects of miR-30c.

### Overexpression of miR-30c attenuates the migratory and invasive phenotypes of ecto-EECs by blocking the BCL9/Wnt/CD44 regulatory cascade

**Fig. 4:**
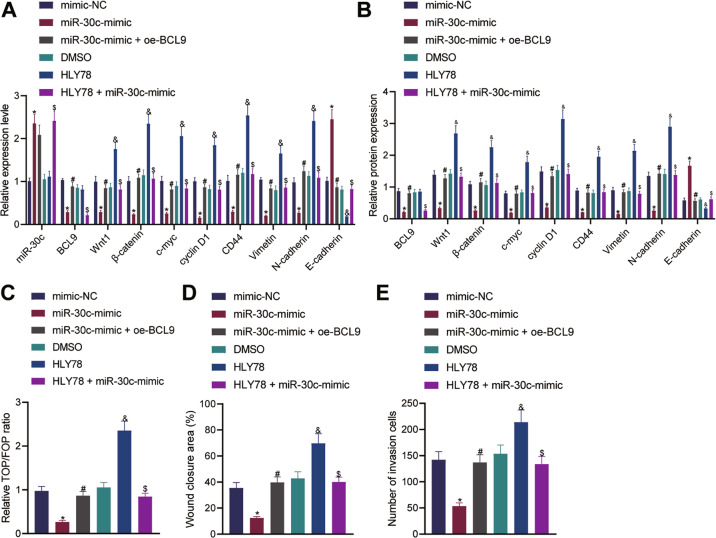
miR-30c attenuated the invasion and migration of ecto-EECs through the BCL9/Wnt/CD44 regulatory cascade. **A** qRT-PCR measurement of the expression of related genes in ecto-EECs of each group; **B** Western blot measurement of the protein expression of related factors in ecto-EECs of each group; **C** Dual-luciferase reporter assay to detect the Wnt gene activity, as reflected by the TOP/FOP ratio, in ecto-EECs of each group; **D** Wound healing assay to detect cell migration in ecto-EECs of each group; **E** Transwell assay to detect cell invasion in ecto-EECs of each group. Cellular experiments were repeated three times. Measurement data were characterized as mean ± SD. The comparison among data of multiple groups was performed with one-way ANOVA with Tukey’s post hoc test. *p < 0.05 versus the mimic-NC group; #*p* < 0.05 versus the miR-30c-mimic group; &*p* < 0.05 versus the DMSO group; $*p* < 0.05 versus the HLY78 group.

Further to examine whether the Wnt/CD44 signaling pathway was the downstream pathway of miR-30c/BCL9, we upregulated miR-30c in ecto-EECs and then confirmed results through qRT-PCR, Western blot, and dual-luciferase assays (Fig. 4A–C). miR-30c-mimic treatment resulted in increased expression of miR-30c and E-cadherin, downregulated expression of BCL9, Wnt1, β-catenin, c-myc, cyclin D1, CD44, Vimentin, and N-cadherin, along with a decrease in the ratio of TOP/FOP (indicating activity of Wnt/β-catenin), collectively indicating inhibition of the Wnt/CD44 signaling pathway. The aforementioned effects of miR-30c restoration alone were reversed by additional overexpression of BCL9. Meanwhile, ecto-EECs treated by HLY78 (a Wnt/β-catenin signaling pathway activator) presented with, as compared with those treated by DMSO, no obvious changes in miR-30c and BCL9 expression yet activation of the Wnt/CD44 signaling pathway, corresponding to upregulated expression of Wnt1, β-catenin, c-myc, cyclin D1, CD44, Vimentin, N-cadherin, and TOP/FOP ratio, as well as reduced E-cadherin. Besides, these effects of HLY78 were abrogated by simultaneous miR-30c-mimic treatment.

Further, we manipulated the expression of miR-30c alone or in combination with BCL9 to assess the effect of miR-30c on ecto-EECs *via* regulation of the BCL9/Wnt/CD44 axis. According to the results (Fig. 4D, E), ecto-EECs of the miR-30c mimic group displayed reduced invasive and migratory potentials, while ecto-EECs overexpressing both miR-30c and BCL9, relative to the former, presented with enhanced cell invasion and migration; meanwhile, miR-30c-mimic treatment was observed to abrogate the stimulating effects of HLY78 treatment alone on ecto-EEC invasion and migration.

Taken together, our data supported that miR-30c overexpression diminished the invasion and migration in ecto-EECs by blocking the BCL9/Wnt/CD44 signaling pathway and that Wnt/β-catenin signaling activator HLY78 could reverse the inhibitory effects of miR-30c.

### miR-30c delivered by EECs-derived exosomes suppressed ecto-EEC metastasis in nude mice

Following the aforementioned cellular experiments, we moved to in vivo substantiation of the findings in an EMs mouse model. On the 14th day after the injection of ectopic endometrial tissue suspension, endometrioid lesions were observed in the intestine, mesentery and peritoneum of mouse models, and adhesions were found around the lesions, suggesting the successful establishment of an EMs mouse model.

**Fig. 5:**
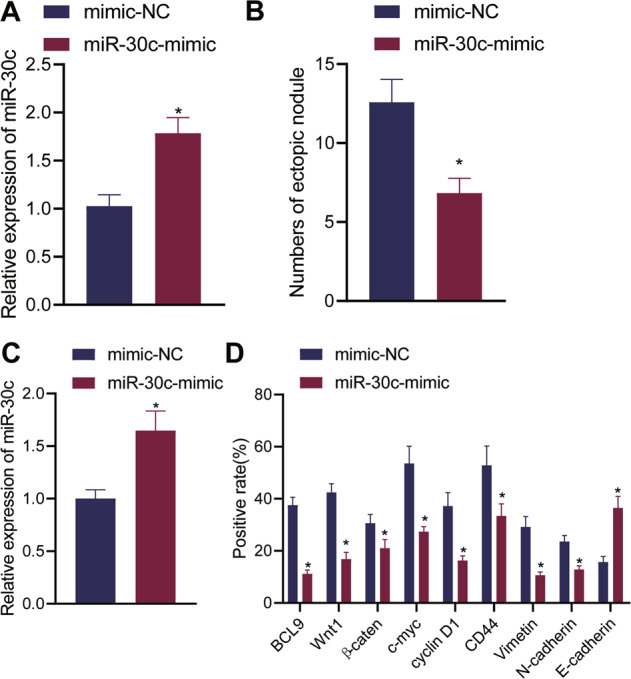
miR-30c delivered by EECs-derived exosomes suppressed ecto-EEC metastasis in nude mice. **A** qRT-PCR measurement of the expression of miR-30c in exosomes derived from ecto-EECs treated with miR-30c-mimic; **B** Detection of EMs metastasis in endometrial tissues of mice on the 14th day from the intraperitoneal injection of exosomes; **C** qRT-PCR measurement of the expression of miR-30c in ectopic endometrial tissue of mice (*n* = 12); **D** Immunohistochemical detection of the expression of related proteins in ectopic endometrial tissues of mice (*n* = 12). Measurement data were characterized as mean ± SD. The comparison between data of two groups was performed by an independent sample *t*-test. *p < 0.05 versus the mimic-NC group.

Subsequently, exosomes were derived from ecto-EECs pretreated with negative control (NC) or miR-30c mimic. qRT-PCR results indicated that the expression of miR-30c in exosomes of the miR-30c mimic group was higher than that in the mimic-NC group (Fig. 5A). Exosomes of the two groups were injected into mice of the corresponding groups, every 2 days for a total of 14 days.

Relatively fewer ectopic nodules were observed in the intestinal wall of EMs mice in the presence of miR-30c overexpression (Fig. 5B). qRT-PCR results confirmed that the endometrial tissue of the miR-30c-mimic-treated mice presented with elevated miR-30c expression (Fig. 5C). Further, it was revealed by immunohistochemistry that the expression of BCL9, Wnt1, β-catenin, c-myc, cyclin D1, CD44, Vimentin, and N-cadherin was reduced while the expression of E-cadherin was increased in response to miR-30c restoration (Fig. 5D).

Collectively, these results illuminated that miR-30c delivered by EECs-derived exosomes attenuated the metastasis of ecto-EEC ectopic nodules *via* the BCL9/Wnt/CD44 regulatory cascade in nude mice.

## Discussion

With an aim to discover new therapeutic targets for EMs, we delineated in the present study a novel mechanism, by which miR-30c attenuated the biological phenotypes of ecto-EECs by blocking the BCL9/Wnt/CD44 regulatory axis in O-EMs.

Our initial finding through bioinformatics analysis indicated the poor expression of miR-30c and the overexpression of BCL9 in O-EMs, which were then validated in clinically collected O-EMs ectopic tissues and ectopic EECs, indicating their involvement in the progression of O-EMs. These findings corroborate a previous study where a downregulated expression has been observed in ectopic and eutopic endometriosis tissues [12]. Meanwhile, although BCL9 has rarely been correlated with EMs, the BCL2 protein family is well-recognized for its regulatory role in relation to endometrial cell apoptosis in EMs [20, 21]. Interestingly, miR-30c has been reported to be poorly expressed in a variety of malignancies, such as colorectal carcinoma, glioblastoma, and urothelial carcinoma, and to exert tumor-suppressive functions by impeding the biological behaviors of cancer cells [22–24]. EMs, even as a relatively benign disease, shares biological behaviors of invasion and migration with cancers [7]. In this study, our results unraveled that exosomes derived from normal EECs suppressed the tumor-like invasive and migratory potentials of ecto-EECs by delivering miR-30c. In relation to our findings, exosome-mediated delivery of miR-30c has been recognized as a diagnostic biomarker or therapeutic target in pancreatic cancer [14], clear cell renal cell carcinoma [13], and myocardial ischemia [25].

Furthermore, our data identified that miR-30c restricted the invasion and migration of ecto-EECs by inversely regulating BCL9, which could be reversed by BCL9 overexpression. Partially consistent with our finding, accumulating evidence has demonstrated that BCL9 was upregulated in numerous malignancies as a consequence of the downregulation of tumor-suppressing microRNAs which negatively mediated BCL9, such as miR-30c-2* in ovarian cancer [26], miR-30a in gastric cancer [27], and miR-1301 in hepatocellular carcinoma [28]. Of note, BCL9 is a well-established transcriptional activator of the Wnt pathway and binds to β-catenin *via* a highly conserved HD2 domain [29, 30], and its oncogenic potential has been ascribed only to the selective binding to β-catenin and thus to its role as an activator of the Wnt/β-catenin signaling pathway [31, 32]. Moreover, CD44 functions as a well-described target gene of the Wnt/β-catenin signaling pathway [19]. Corroborating the aforementioned evidencee, our data illuminated that miR-30c overexpression diminished the invasion and migration in ecto-EECs by blocking the BCL9-mediated activation of the Wnt/CD44 axis and that HLY78, an activator of the Wnt/β-catenin signaling pathway, could reverse the inhibitory effects of miR-30c. Following in vitro findings, our in vivo experiments showed that miR-30c delivered by EECs-derived exosomes attenuated the metastasis of ecto-EEC ectopic nodules through modulation of the BCL9/Wnt/CD44 regulatory axis. In relation to our findings, Ling, X. H. et al. have correlated the ectopic expression of miR-30c with repressed expression of Wnt pathway downstream targets, including CD44 and c-Myc in prostate cancer cells; they also observed upregulated expression of BCL9 in prostate tissues and identified that BCL9 was targeted by miR-30c [33]. Moreover, another prior report has suggested that miR-30 can act as a tumor suppressor by modulating the oncogenic Wnt/β-catenin/BCL9 pathway in a broad range of human cancers [15].

**Fig. 6:**
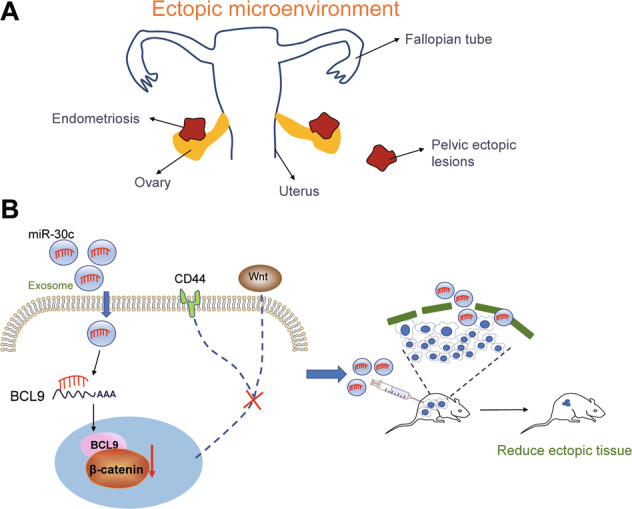
The mechanism graph of the regulatory network and function of miR-30c. **A** The microenvironment of ovarian endometriosis; **B** The mechanism graph of the regulatory network and function of miR-30c. miR-30c delivered by EECs-derived exosomes inversely regulated BCL9 expression, and blocked the Wnt/β-catenin signaling pathway along with the downstream CD44 expression, thus contributing to the suppression of the invasion and migration of ecto-EECs in EMs.

On the basis of the evidence acquired in the present study, it can be concluded that exosomal miR-30c repressed the biological phenotypes of ecto-EECs in O-EMs through the miR-30c/BCL9/Wnt/CD44 regulatory cascade. Specifically, miR-30c delivered by EECs-derived exosomes could inversely regulate BCL9 expression and thus block the Wnt/β-catenin signaling pathway along with the downstream CD44, which repressed the invasion and migration of ecto-EECs in O-EMs (Fig. 6). By elucidating the mechanism underlying the suppressive role of miR-30c in EMs, this study deepened our understanding of the etiology of O-EMs and provides novel potential therapeutic targets for the treatment of O-EMs.

## Methods and materials

### Ethics statement

This study was approved by the Institutional Ethics Review Board of the Second Xiangya Hospital, Central South University (#2018-181). All experiments were conducted in strict accordance with the principles of the Helsinki Declaration. Written informed consents were signed and submitted by each participant. Animal experiments were approved by the Animal Care and Use Committee of the Second Xiangya Medical College of Central South University and performed in accordance with *Guide for the Care and Use of Laboratory Animals* published by the National Institutes of Health.

### Tissue collection

Matched eutopic and ectopic endometrial tissue samples were collected from a total of 24 patients (aged 21 to 43 years) who underwent laparoscopic detection for O-EMs in the Second Xiangya Hospital, Central South University from June 2019 to March 2020 and later pathologically diagnosed with EMs. According to pathological observation and/or information on patients’ menstrual period, all tissue samples were in the middle and late stages of proliferation. All patients were diagnosed as stage III/IV on the basis of the Revised American Fertility Society Classification of Endometriosis. Patients who received any hormone therapy or more than one year of contraception before the surgery were excluded from the study, and those presented with no symptoms in ovaries, fallopian tubes or uterus were also excluded. Meanwhile, the normal endometrial samples (*n* = 20) of the control group were harvested from patients with infertility related to fallopian tube pathology in the same period. Part of the tissue samples were fixed with formalin, and the other part were stored at −80 °C.

### High-throughput transcriptome sequencing

A total of five pairs of eutopic and ectopic endometrial tissues of O-EMs patients were subjected to the extraction of total RNA following the protocols of Trizol reagent (Invitrogen, Carlsbad, CA). The concentration of the RNA was then determined by Nanodrop ND-1000 spectrophotometer (Thermo Fisher Scientific, San Jose, CA) with the optical density (OD) measured at 260/280 wavelength, and the integrity of RNA was detected by agarose gel electrophoresis (28 s/18 s ratio >1.5). After that, the An Illumina TruSeq^®^ Small RNA Library Prep Kit was utilized for miRNA sequencing, and a total of 5 µg RNA from each sample was detected, followed by quantification and quality control using the KAPA Library Quantitative Kit (KAPA Biosystems, Woburn, MA). Then, paired-end sequencing was performed on the Illumina NextSeqCN500 sequencer, and differentially expressed miRNAs related to O-EMs were screened out through the high-throughput transcriptome sequencing of the eutopic and ectopic endometrial tissue samples from O-EMs patients.

### Bioinformatics analysis

An EMs-related microarray containing five tissue samples of O-EMs patients and five of non-EMs patients was retrieved from the Gene Expression Omnibus (GEO) database. Differentially expressed genes (DEGs) in O-EMs samples were then identified utilizing the Limma package in R language, with *p* < 0.05 as the screening threshold.

### Isolation and culture of primary endometrial epithelial cells

EMs ectopic endometrial epithelial cells (ecto-EECs), EMs eutopic endometrial epithelial cells (euto-EECs), and normal human endometrial epithelial cells (normal EECs) were isolated from clinically collected O-EMs tissues (within 2 h after the collection). Briefly, PBS-washed tissue samples were cut into small pieces and incubated with 0.25% type IV collagenase-trypsin EDTA (three times the volume of tissue pieces; Sigma, St. Louis, MO) at 37 °C. Following trypsinization, the tissue solution was filtered through 150 and 74 μm filters, and cells obtained were cultured in DMEM/F-12 medium supplemented with 10% FBS and 1% antibiotic-antifungal solution (Gibco, Gaithersburg, MD). All cellular experiments were performed before the fourth cell passage.

### Cell transfection

The cells were transfected with plasmids carrying miR-30c mimic alone or in combination with BCL9 overexpression plasmids (oe-BCL9), miR-30c inhibitor, or corresponding NC, labeled as miR-30c-mimic, miR-30c-mimic + oe-BCL9, miR-30c-inhibitor, inhibitor-NC, and mimic-NC groups, respectively. Specifically, cells were seeded into six-well plates (1 × 10^5^ cells/well) and incubated, upon the cell confluence reaching 60−70%, with optimem medium (750 μL/well). Then, 5 μL Lipo3000 reagent (L3000001, Invitrogen) in 120 μl optimum was mixed with the aforementioned plasmids loaded in 125 μL of optimem and allow to stand for 20 min. The mixture (250 μL) was added to cells in each well, followed by incubation with the medium updated every 16 h. Total RNA was extracted from the cells 48 h later, and total protein was extracted 72 h later.

### qRT-PCR

Based on the protocols of the mirVanaTM PARISTM RNA kit (AM1556, Invitrogen), miRNA was isolated from exosomes, tissues, and cells. Total RNA (1 µg) was reversely transcribed into cDNA utilizing the First Strand cDNA Synthesis Kit (K1622, Fermentas) for mRNA analysis, while the cDNA for miRNA analysis was synthesized with a polyA-tailing MicroRNA Reverse Transcription Kit (EZB-miRT2-S, EZBioscience, Roseville, MN). After that, the Fast SYBR Green PCR kit (Applied Biosystems) and ABI PRISM 7300 RT-PCR system (Applied biosystems) were employed for the qRT-PCR assay, and three replicate wells set for each sample. U6 was used as a housekeeper gene for miR-30c, and GAPDH was for other genes. Further, the 2^−ΔΔCt^ method was used to calculate the relative transcription level of target genes. Involved primers were commercially obtained (Sangon Biotech, Shanghai, China), as listed in Supplementary Table 1.

### Western blot assay

Total protein was extracted from cells utilizing RIPA lysis (Beyotime, Shanghai, China) supplemented with protease inhibitor and phosphorylase inhibitor, followed by measurement of protein concentration utilizing a BCA protein assay kit (P0010S, Beyotime). Afterward, the protein solution was added with 5× loading buffer (Beyotime) and boiled at 100 °C for 10 min for denaturation. Protein (20 µg for each sample) was separated by sodium dodecyl sulfate polyacrylamide gel electrophoresis, electro-transferred to polyvinylidene fluoride membrane, and blocked with 5% BSA for 2 h at room temperature to suppress nonspecific binding. Subsequently, the cells were incubated overnight at 4 °C with corresponding diluted primary antibodies (Supplementary Table 2), followed by 1-h incubation with HRP-labeled IgG secondary antibody (1:2000, ZSGB-BIO, Beijing, China). Protein bands were then visualized utilizing ECL solution (BM101, Biomiga, San Diego, CA) and BioSpectrum 600 imaging system (Ultra-Violet Products, Cambridge, UK). The gray level of protein bands was quantified with the ImageJ analysis software (normalized to GAPDH).

### Transwell invasion assay

The Transwell system was precoated with Matrigel (356235, BD Biosciences, Bedford, MA). Cells in the logarithmic growth phase were incubated in serum-free DMEM medium for 24 h, digested, and resuspended to reach a final concentration of 1 × 0^5^ cells/ml. Then, 200 μL of the cell suspension was added to the upper chamber of the Transwell system while pre-chilled DMEM medium (containing 10% FBS) was added to the lower chamber. After 48-h incubation, the cells were subjected to 10-min cell fixing using 4% paraformaldehyde and staining utilizing 0.5% crystal violet. Cells penetrating the basement membrane were observed and counted in five seldomly selected fields of view under an inverted microscope.

### Wound healing assay

A total of 1 × 10^6^ cells were seeded in six-well plates for incubation at 37 °C. When a monolayer of cells occurred, a pipette tip was used to make scratches in the central region, followed by cell incubation. With the time point of making scratches set as 0 h, the cells were observed at 12, 24, and 48 h, and images were photographed at 48 h. The ImageJ software was then utilized to analyze the images and calculate the wound healing rate.

### Exosome isolation

The exosomes in the culture medium of normal or ectopic endometrial cells were isolated and purified by ultracentrifugation. The cells were cultured in exosome-free DMEM/F-12 medium for 48 h. and the culture medium was collected for 20-min centrifugation (2 × 10^3^ g) at 4 °C to remove cell debris, followed by a 30-min centrifugation (1 × 10^4^ g) at 4 °C to remove cell debris. The supernatant was collected and filtered through a 0.2 μm filter (micropore) and then subjected to 1-h ultracentrifuged (1 × 10^5^ g) at 4 °C. The precipitate was resuspended in PBS and again subjected to 1-h ultracentrifuged (1 × 10^5^ g). After that, separated exosomes were collected from the supernatant using exosome precipitation reagent (EXOTC10A-1, YUBO Biological, Shanghai, China).

### TEM

Isolated exosomes were subjected to TEM observation and images were photographed. The exosomes were resuspended, fixed with 30 μL of 2% paraformaldehyde, and then transferred to the discharged copper grid. The copper grid with the exosomes was immersed in 3% glutaraldehyde for fixation, and then 4% uranyl acetate was added dropwise to stain the exosomes, followed by observation using TEM (FEI, Hillsboro, OR).

### Fluorescence staining of exosomes and confocal microscopy

Exosomes derived from normal endometrial cells were stained with PKH26 (MINI26-1KT) for 2 h. The labeled exosomes (red) were cocultured with ecto-EECs cells for 4 h, followed by observation of the coculture system using a confocal microscope (FV1200, Olympus, Hamburg, Germany).

### Dual-luciferase reporter gene assay

The HEK-293T cell line (purchased from the cell bank of Shanghai Institute of Cells, Chinese Academy of Sciences) was cultured in a DMEM medium. When the cell confluence reached 80−90%, the cells were digested with 0.25% trypsin, passaged, and routinely cultured (5% CO_2_, 37 °C). Target genes of miR-30c were predicted utilizing the targetscan.org website, and the dual-luciferase reporter assay was conducted to verify whether BCL9 was the direct target.

The BCL9 3′UTR sequence containing the miR-30c binding site was inserted into the psiCHECK-2 luciferase reporter gene vector (Promega, Madison, WI) to construct BCL9-WT (UCUCCCA); and the BCL9 3′UTR sequence was mutated using the SiteDirected mutagenesis kit (TransGene, Beijing, China) for the construction of the BCL9-MUT (AGAGGGU) in the same manner. Next, HEK-293T cells were seeded into 24-well plates for 24-h incubation and then transfected with BCL9-WT or BCL9-MUT constructs and miR-30c mimic or mimic-NC. The relative luciferase activity was evaluated using a dual-luciferase reporter system (E1910, Promega) 24 h after the transfection.

### TOP/FOP luciferase activity detection

The cells were transfected with 1.0 µg TOP flash or FOP flash plasmid, 0.1 µg TKRenilla control vector (Promega), and 100 pM plasmids expressing miR-30c (or NC) mimic. Following 24-h transfection, the cells were lysed and the luciferase activity was measured using the dual-luciferase reporter system (Promega).

### Establishment of an EMs nude mouse model

Twenty-four Balb/c female nude mice (aged 6–8 weeks) were housed in a SPF environment (12 h/12 h light/dark cycle, 23−25 °C) and acclimated for 2 weeks. Blocks of clinically collected ectopic endometrial tissues were resuspended with PBS, and the suspension was injected into anesthetized nude mice through the abdominal cavity using 19-gauge needles (0.2 mL PBS containing ~40 mg tissues for each mouse).

Further, 14 days after the injection, the mice were randomly divided into 2 groups (*n* = 12). Mice of one group was injected with miR-30c-loaded exosomes derived from ecto-EECs cells previously treated with miR-30c-mimic through the tail vein, every 2 days until the 14th day (about 100 μg exosomes were injected in total). Mice of the other group, as the control, were injected with exosomes derived from mimic-NC-treated ecto-EECs. During the experiment, all nude mice survived, and no significant difference was observed between the growth rate of mice of the two groups. The mice were euthanized 24 h after the last injection, and the endometrial tissues were collected for subsequent experiments.

### Immunohistochemistry

Paraffin-embedded sections of mouse tissues (store at 60 °C for 2 h) were deparaffinized, hydrated, and immersed in citric acid buffer (0.01 mol/L, pH 6.0) with 30-min heating at 95 to 100 °C. After PBS washing, the sections were incubated with 0.5% TritonX100 for 30 min, stained with the biotin-streptavidin HRP detection system (ZSGB-BIO), and then incubated with corresponding primary antibodies (Supplementary Table 2) overnight at 4 °C. The brown stain on the membrane indicated a positive immunoreaction. The staining was observed using the Nikon ECLIPSE Ti microscope (Nikon, Fukasawa, Japan) and images were analyzed with Nikon software.

### Statistical analysis

Data were processed by SPSS 21.0 software (IBM Corp., Armonk, NY) and GraphPad Prism7. Measurement data were characterized as mean ± SD. The comparison between data of two groups was performed by independent sample *t*-test, and that among multiple groups was performed with one-way analysis of variance (ANOVA) with Tukey’s post hoc test. Pearson correlation was used to analyze the correlation between miR-30c and BCL9. Moreover, *p* < 0.05 was considered as statistically significant difference.

## Supplementary information

Supplementary Tables

full and uncropped western blots

## Data Availability

The data of the study can be obtained from the corresponding author upon request.

## Materials availability

The materials of the study can be obtained from the corresponding author upon request
