# Supplementary material for: Endometrial epithelial cells-derived exosomes deliver microRNA-30c to block the BCL9/Wnt/CD44 signaling and inhibit cell invasion and migration in ovarian endometriosis
Source: Cell Death Discov. 2022 Apr 2;8:151. doi: 10.1038/s41420-022-00941-6 (PMC8976844; doi:10.1038/s41420-022-00941-6)
Supplement: Supplementary file 1 — Supplementary Tables [file 41420_2022_941_MOESM1_ESM.docx]

**Supplementary Table 1** Primer sequences for RT-qPCR

| Genes | Primer sequence |
| --- | --- |
| miR-30c | Forward: 5’-ACCATGCTGTAGTGTGTGTA -3’ |
|  | Reverse: Universal reverse primer |
| BCL9 | Forward: 5’-GGCCATACCCCTAAAGCACTC-3’ |
|  | Reverse: 5’-CGGAAATACTTCGCTCCCTTTT-3’ |
| Wnt1 | Forward: 5’-CGATGGTGGGGTATTGTGAAC-3’ |
|  | Reverse: 5’-CCGGATTTTGGCGTATCAGAC-3’ |
| β-catenin | Forward: 5’-AGTTACTTCACTCTAGGAATGA-3’ |
|  | Reverse: 5’-TCAACTGAAAGCCGTTTCTTGTAA-3’ |
| c-myc | Forward: 5’-GGCTCCTGGCAAAAGGTCA-3’ |
|  | Reverse: 5’-CTGCGTAGTTGTGCTGATGT-3’ |
| cyclin D1 | Forward: 5’-GCTGCGAAGTGGAAACCATC-3’ |
|  | Reverse: 5’-CCTCCTTCTGCACACATTTGAA-3’ |
| CD44 | Forward: 5’-CTGCCGCTTTGCAGGTGTA-3’ |
|  | Reverse: 5’-CATTGTGGGCAAGGTGCTATT-3’ |
| Vimentin | Forward: 5’-AGTCCACTGAGTACCGGAGAC-3’ |
|  | Reverse: 5’-CATTTCACGCATCTGGCGTTC-3’ |
| N-cadherin | Forward: 5’-TCAGGCGTCTGTAGAGGCTT-3’ |
|  | Reverse: 5’-ATGCACATCCTTCGATAAGACTG-3’ |
| E-cadherin | Forward: 5’-CGAGAGCTACACGTTCACGG-3’ |
|  | Reverse: 5’-GGGTGTCGAGGGAAAAATAGG-3’ |
| GAPDH | Forward: 5’-GATTCCACCCATGGCAAATTCC-3’ |
|  | Reverse: 5’-TCGCTCCTGGAAGATGGTGAT-3’ |
| U6 | Forward: 5’-GCAGCACATATACTAAAATTGGAAC -3’ |
|  | Reverse: Universal reverse primer |

Note: RT-qPCR, reverse transcription-quantitative polymerase chain reaction; miR-30c, microRNA-30c; BCL9, B-cell lymphoma 9; CD44, cluster of differentiation 44; GAPDH, glyceraldehyde-3-phosphate dehydrogenase

**Supplementary Table 2** Antibodies used for Western blot assay and immunohistochemistry

| Antibodies | Molecular weight | Dilution rate | Source and product code |
| --- | --- | --- | --- |
| BCL9 | 149kDa | 0.736111 | Proteintech, 22947-1-AP |
| β-catenin | 92kDa | 0.736111 | CST, #8480 |
| Cyclin D1 | 36kDa | 0.736111 | CST, #55506 |
| C-myc | 57kDa | 0.736111 | Proteintech, 67447-1-IP |
| CD44 | 82kDa | 0.736111 | Proteintech, 15675-1-AP |
| Vimentin | 57kDa | 0.736111 | CST, #5741 |
| N-cadherin | 140kDa | 0.736111 | CST, #13116 |
| Wnt1 | 41kDa | 0.736111 | Abcam, ab15251 |
| E-cadherin | 135kDa | 0.736111 | CST, #14472 |
| GAPDH | 37kDa | 0.736111 | CST, #2118 |
| Anti-rabbit IgG (secondary antibody) | | 0.736111 | CST, #7074 |
| Anti-mouse IgG (secondary antibody) | | 0.736111 | CST, #7076 |
| Anti-rabbit IgG (fluorescence) | | 0.388889 | CST, #2985 |
| Anti-mouse IgG (fluorescence) | | 0.388889 | CST, #4417 |

Note: miR-30c, microRNA-30c; BCL9, B-cell lymphoma 9; CD44, cluster of differentiation 44; GAPDH, glyceraldehyde-3-phosphate dehydrogenase; IgG, immunoglobulin G
