# Supplementary figures and images for: Endometrial epithelial cells-derived exosomes deliver microRNA-30c to block the BCL9/Wnt/CD44 signaling and inhibit cell invasion and migration in ovarian endometriosis
Source: Cell Death Discov. 2022 Apr 2;8:151. doi: 10.1038/s41420-022-00941-6 (PMC8976844; doi:10.1038/s41420-022-00941-6)

fig2C


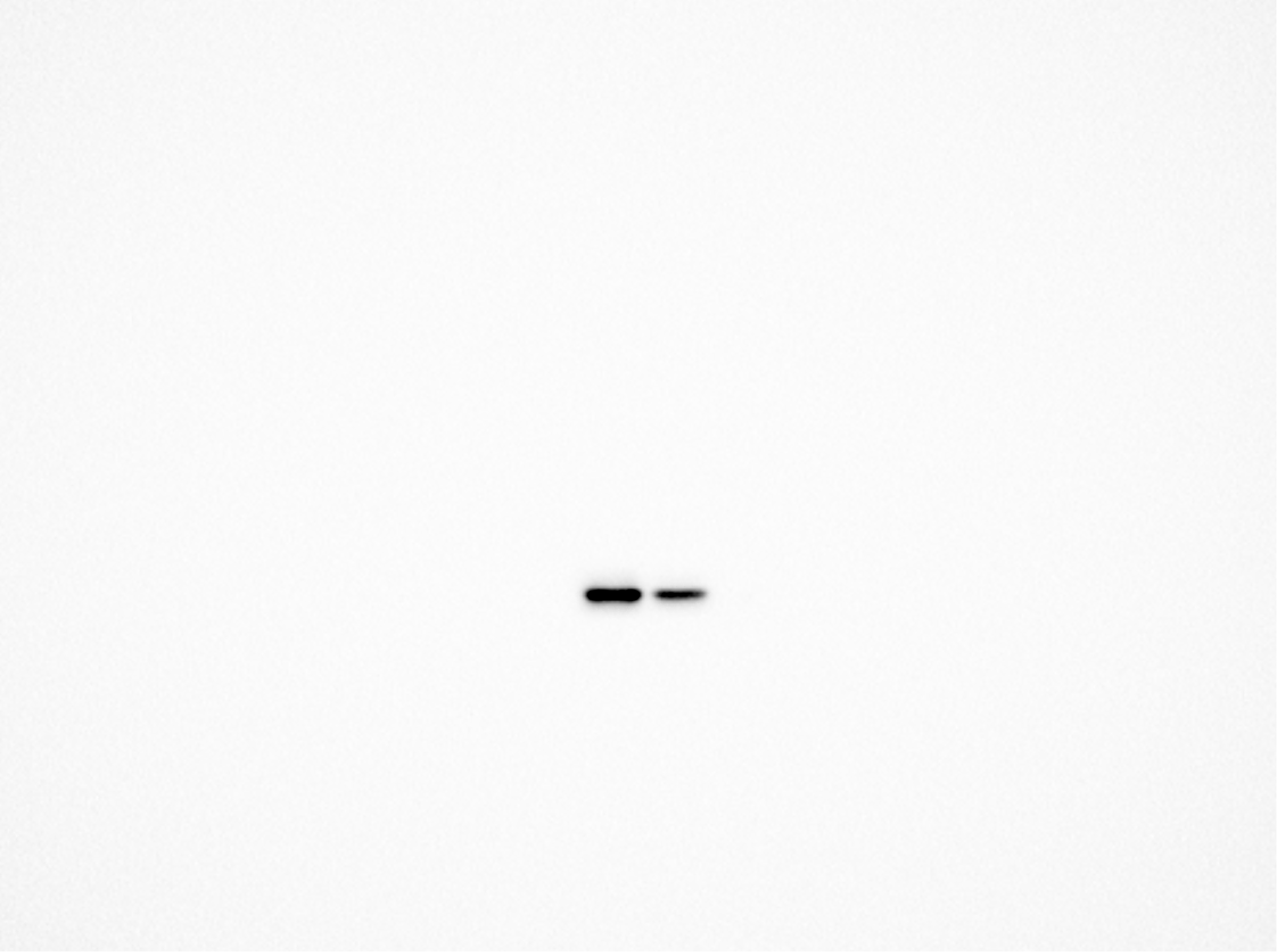


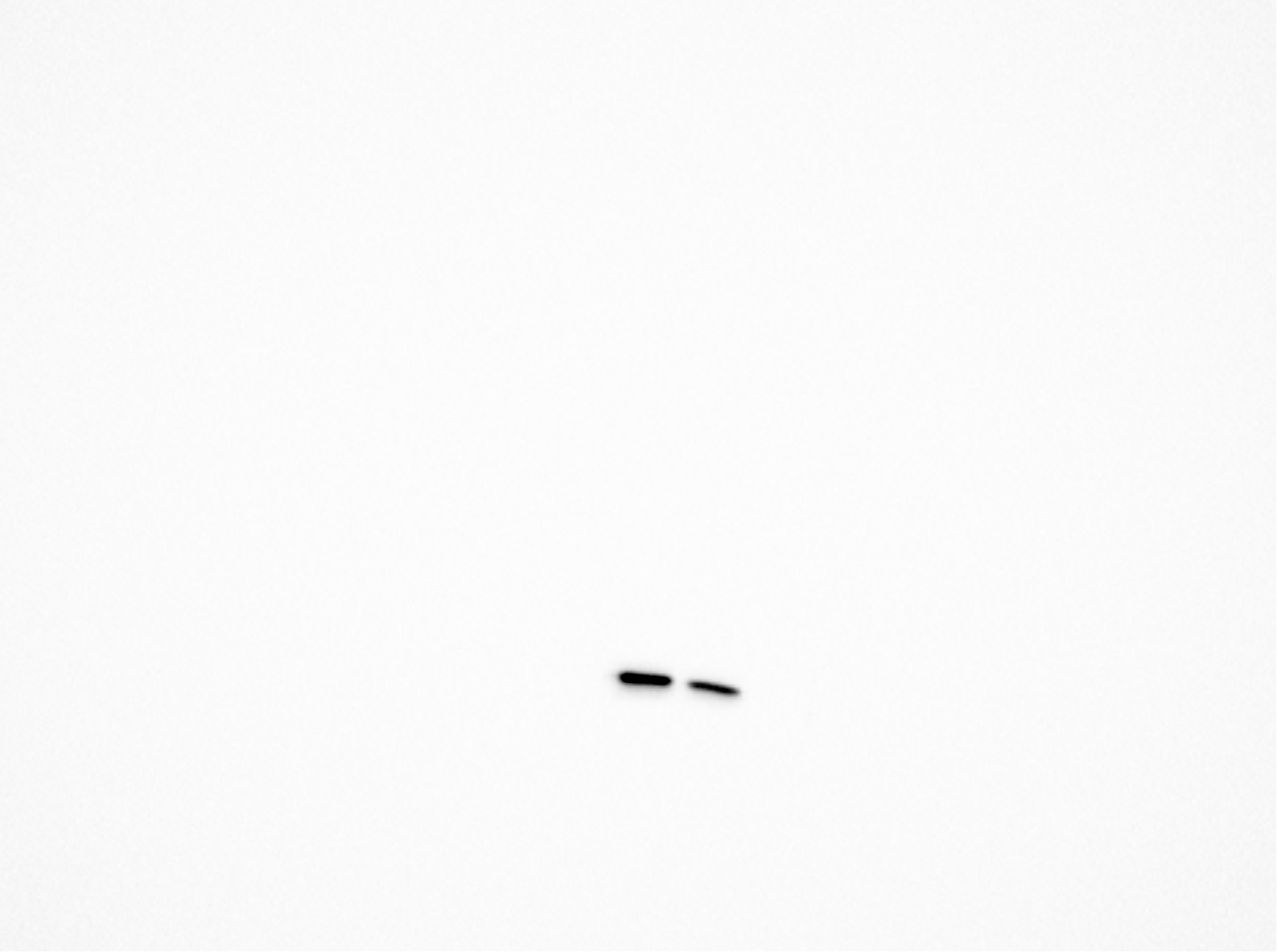


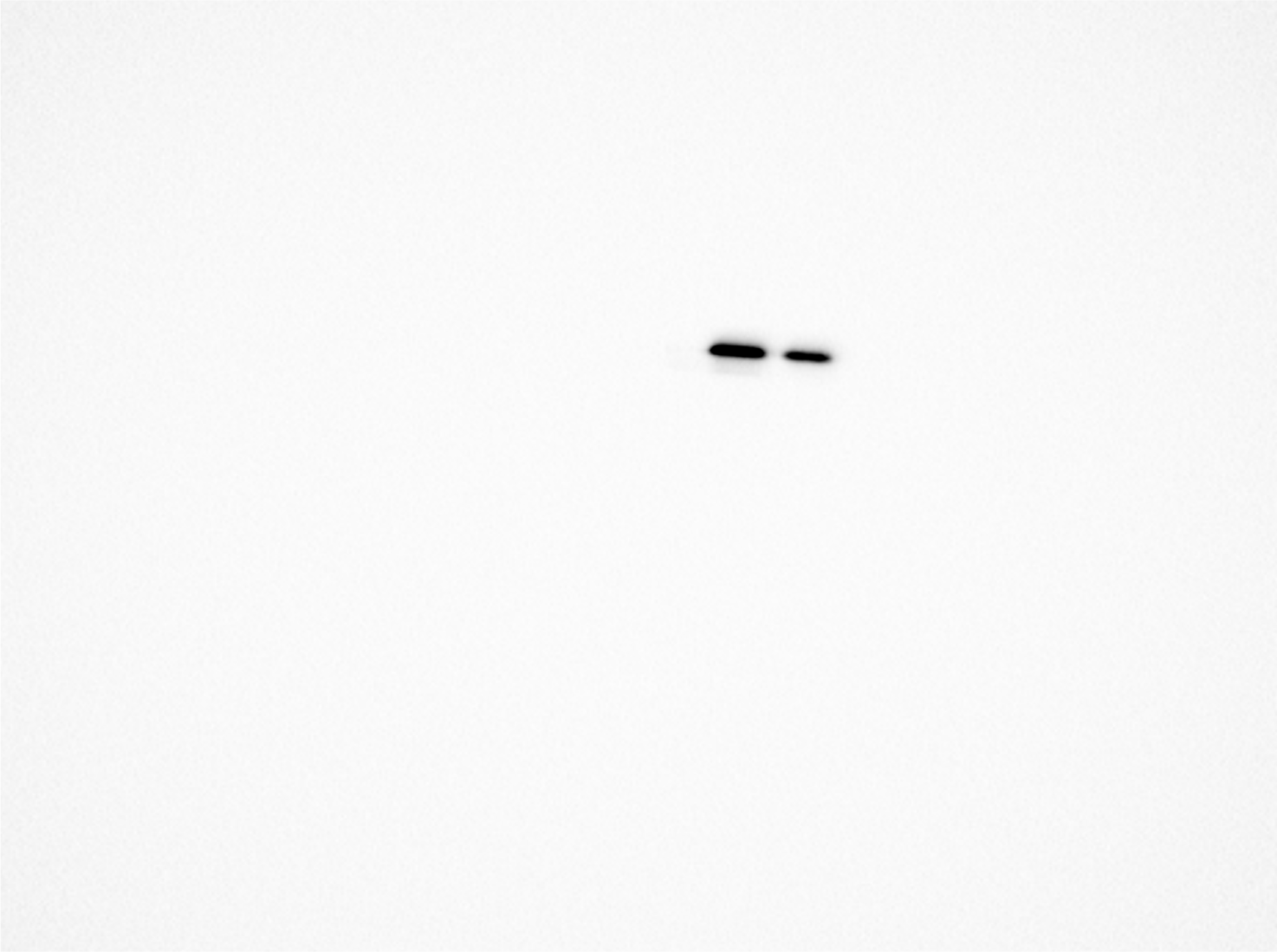


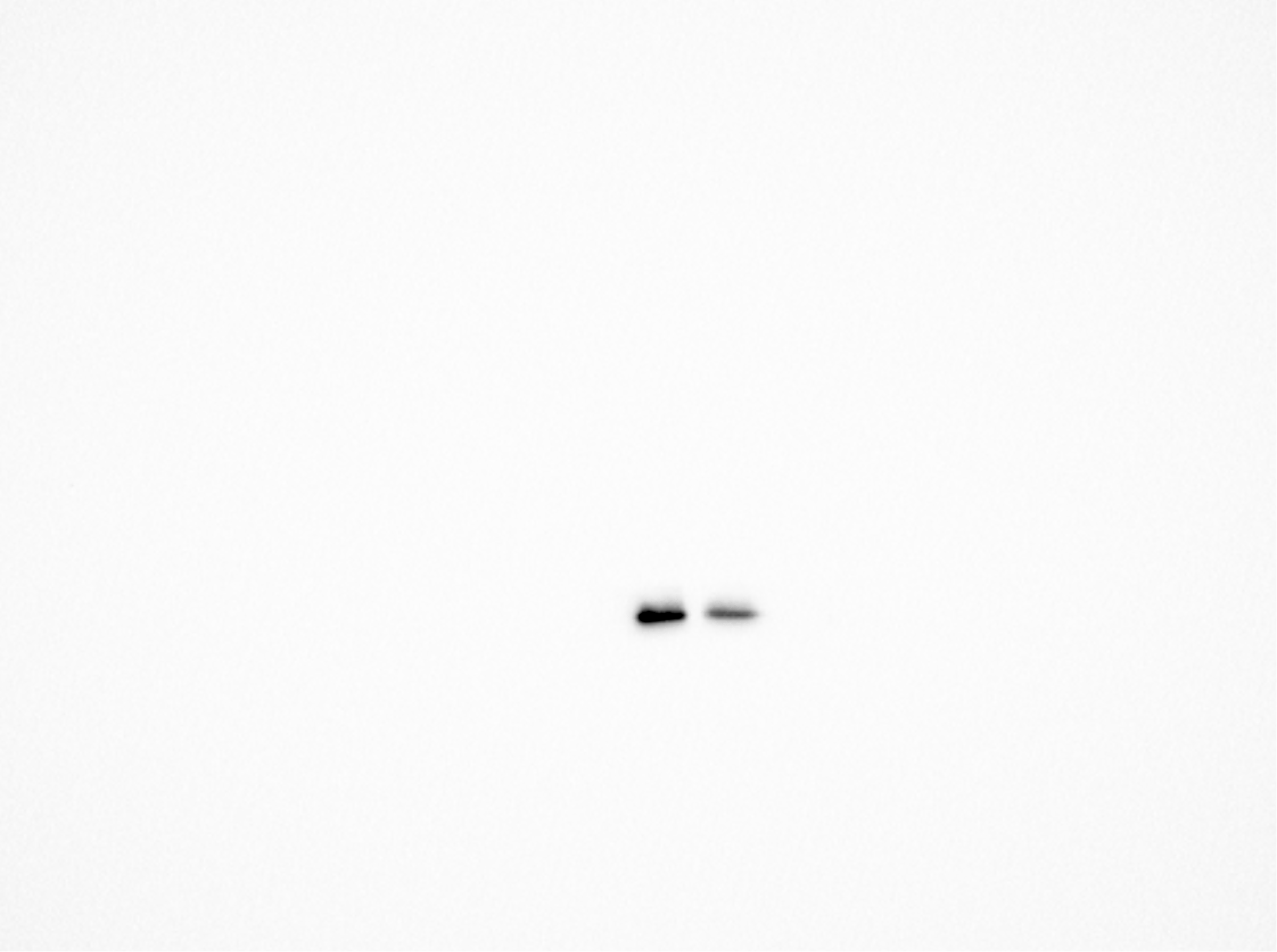


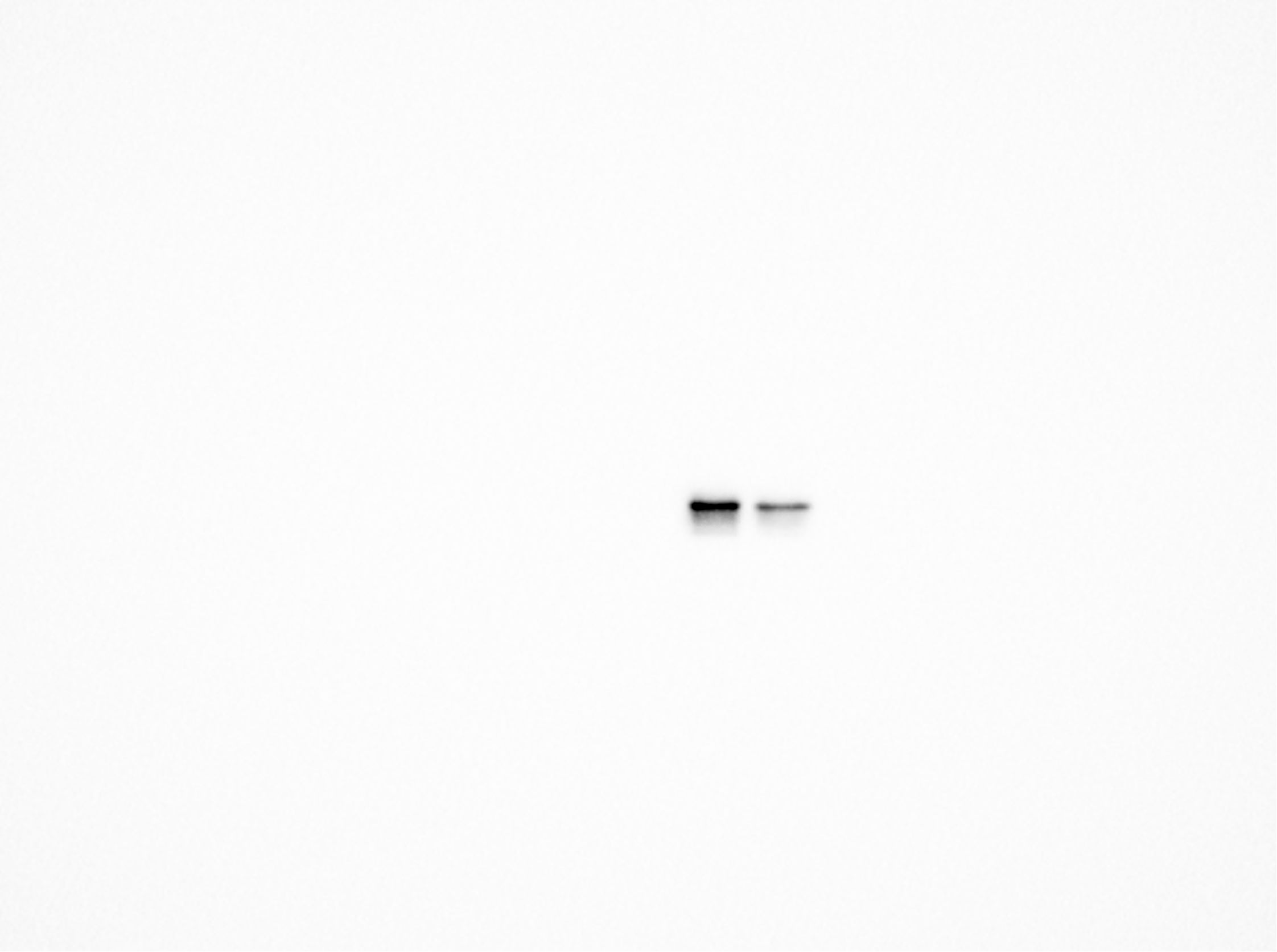


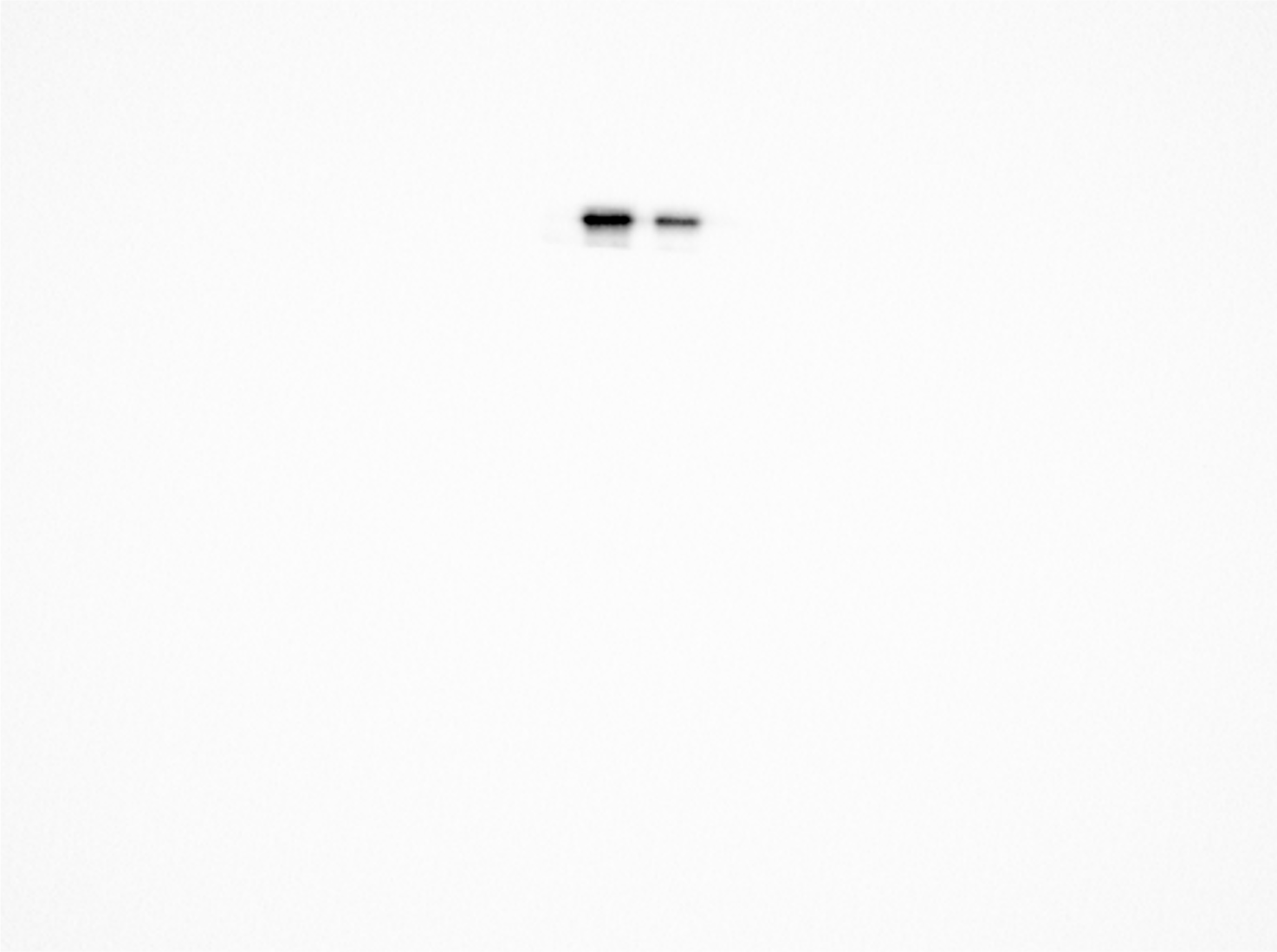


fig2M


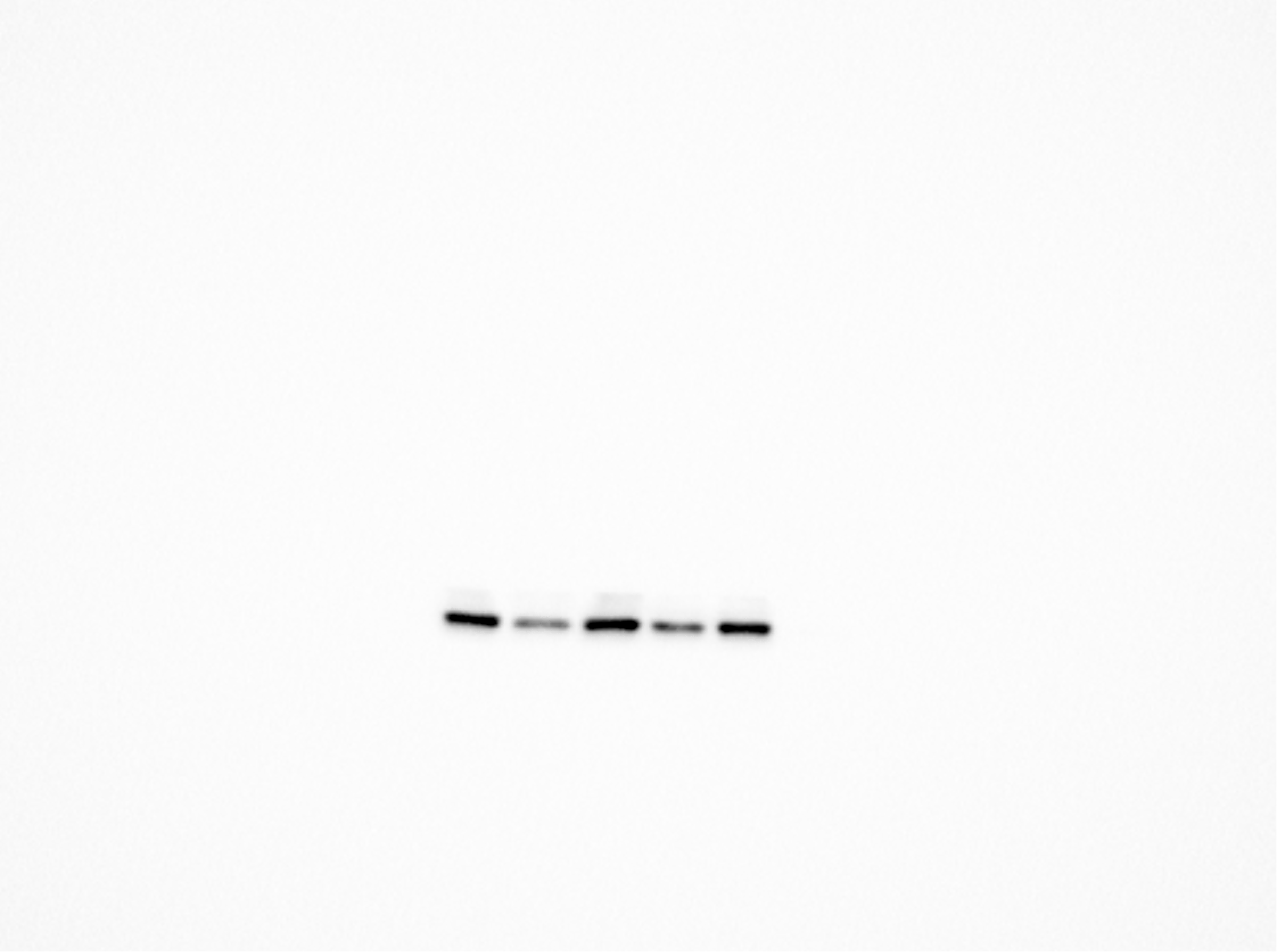


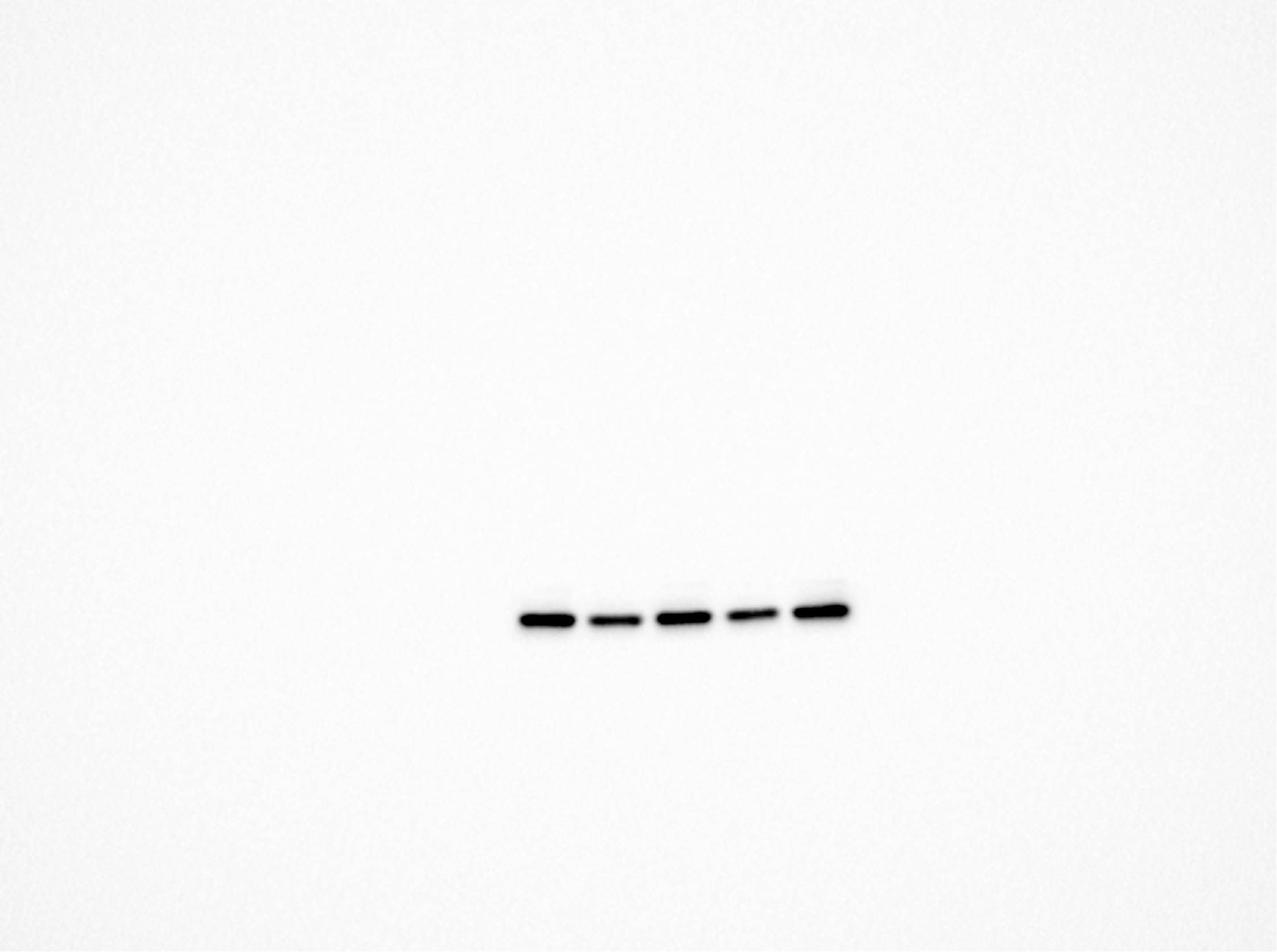


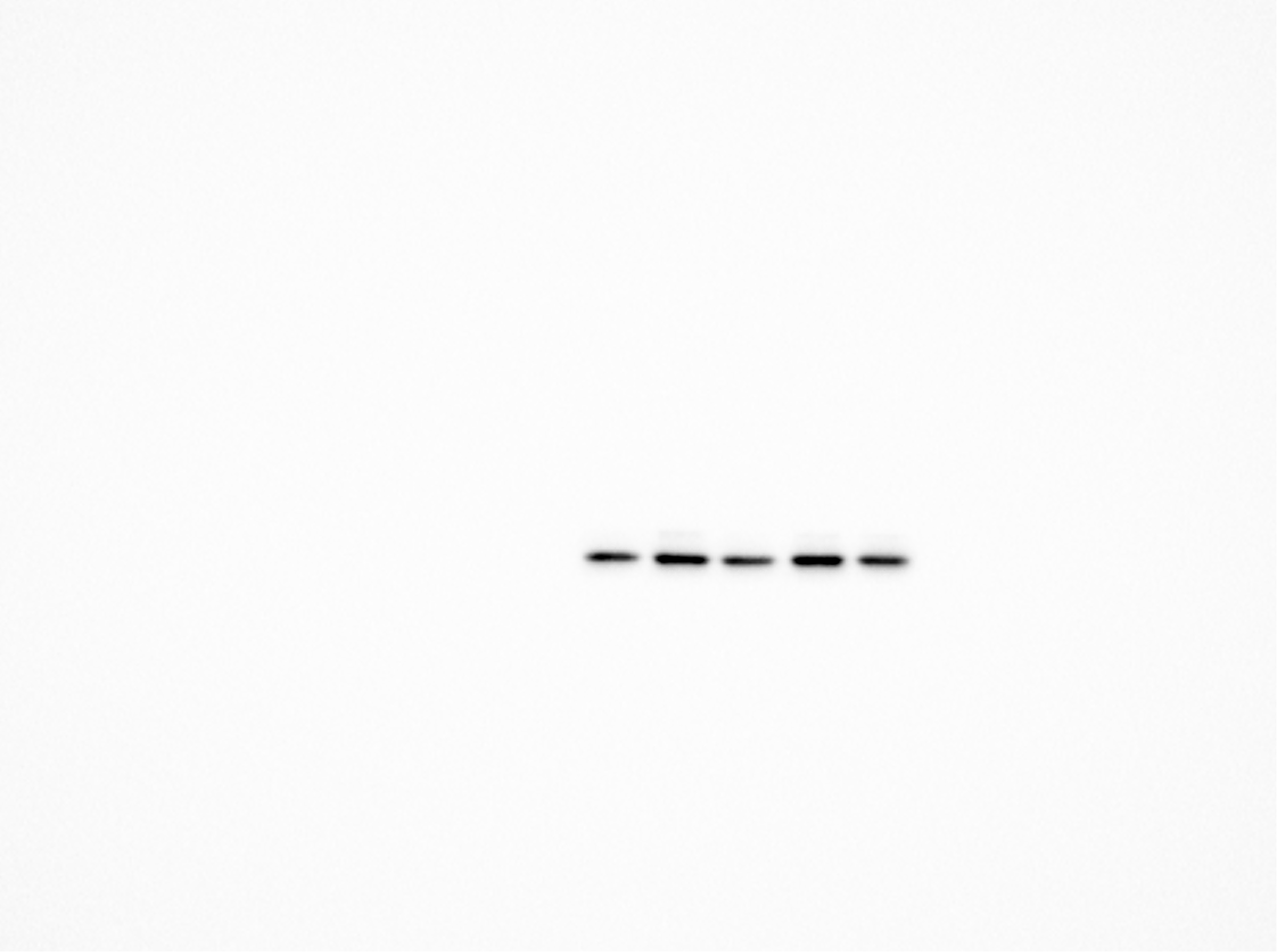


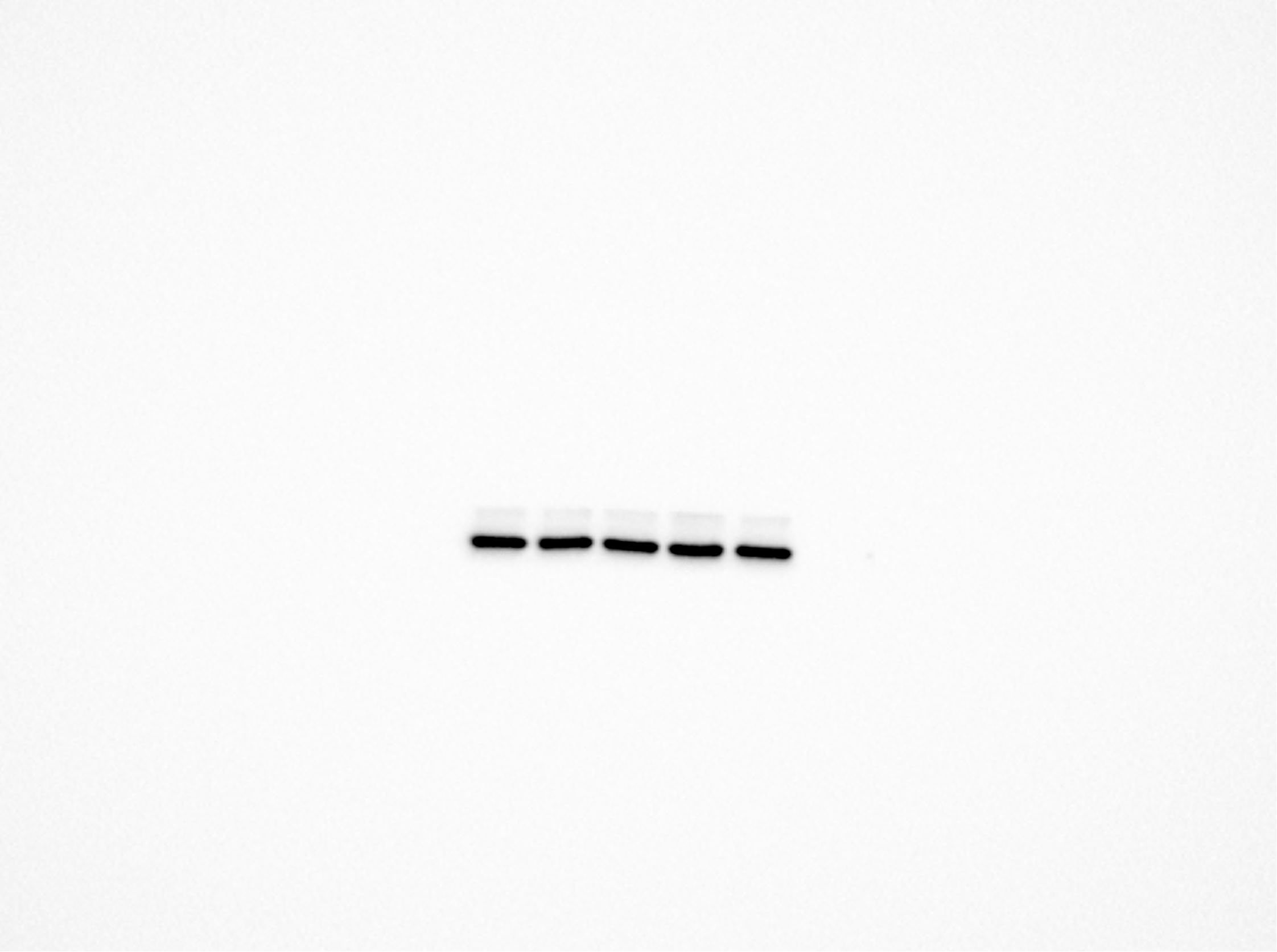


fig3D


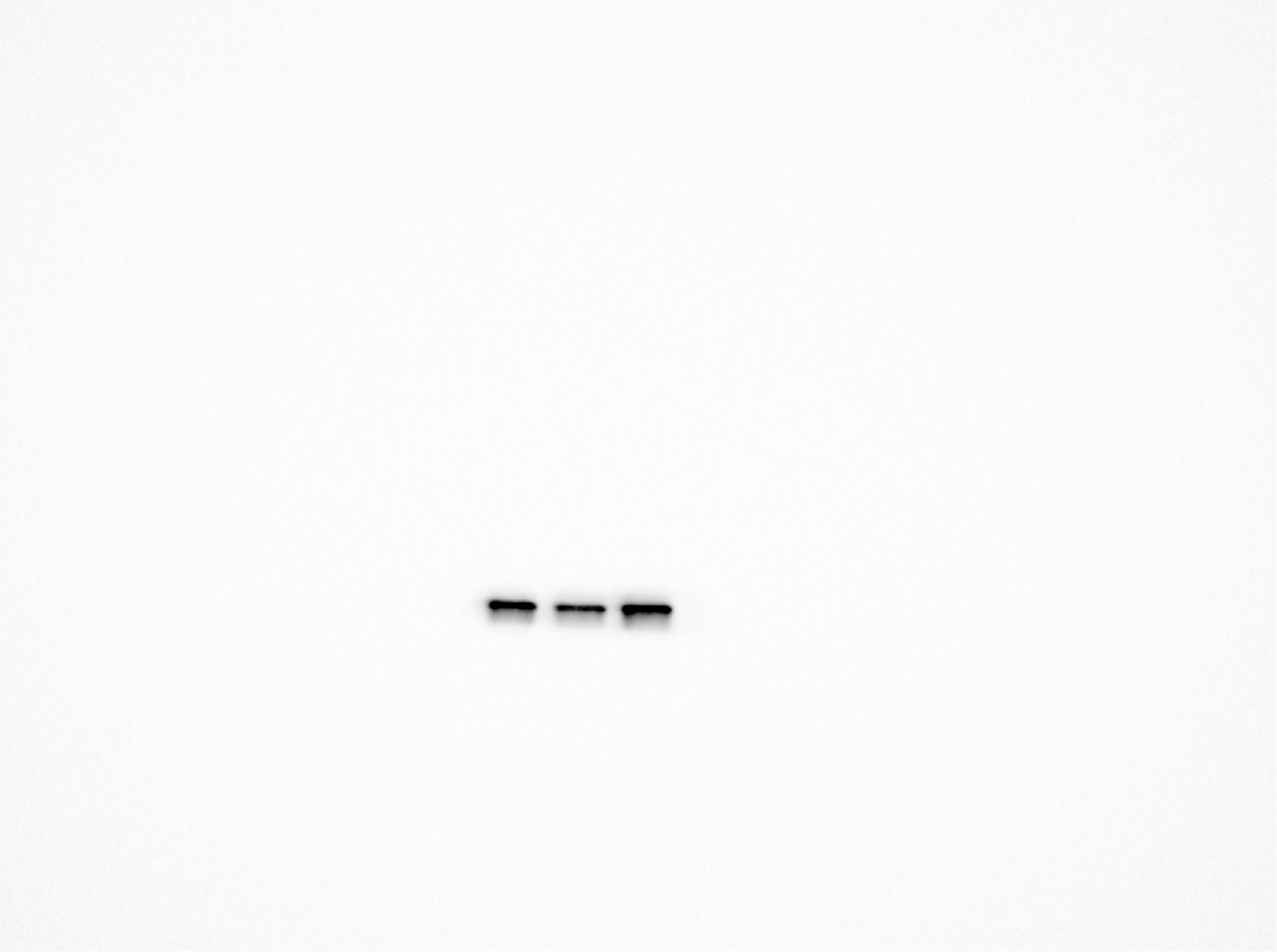


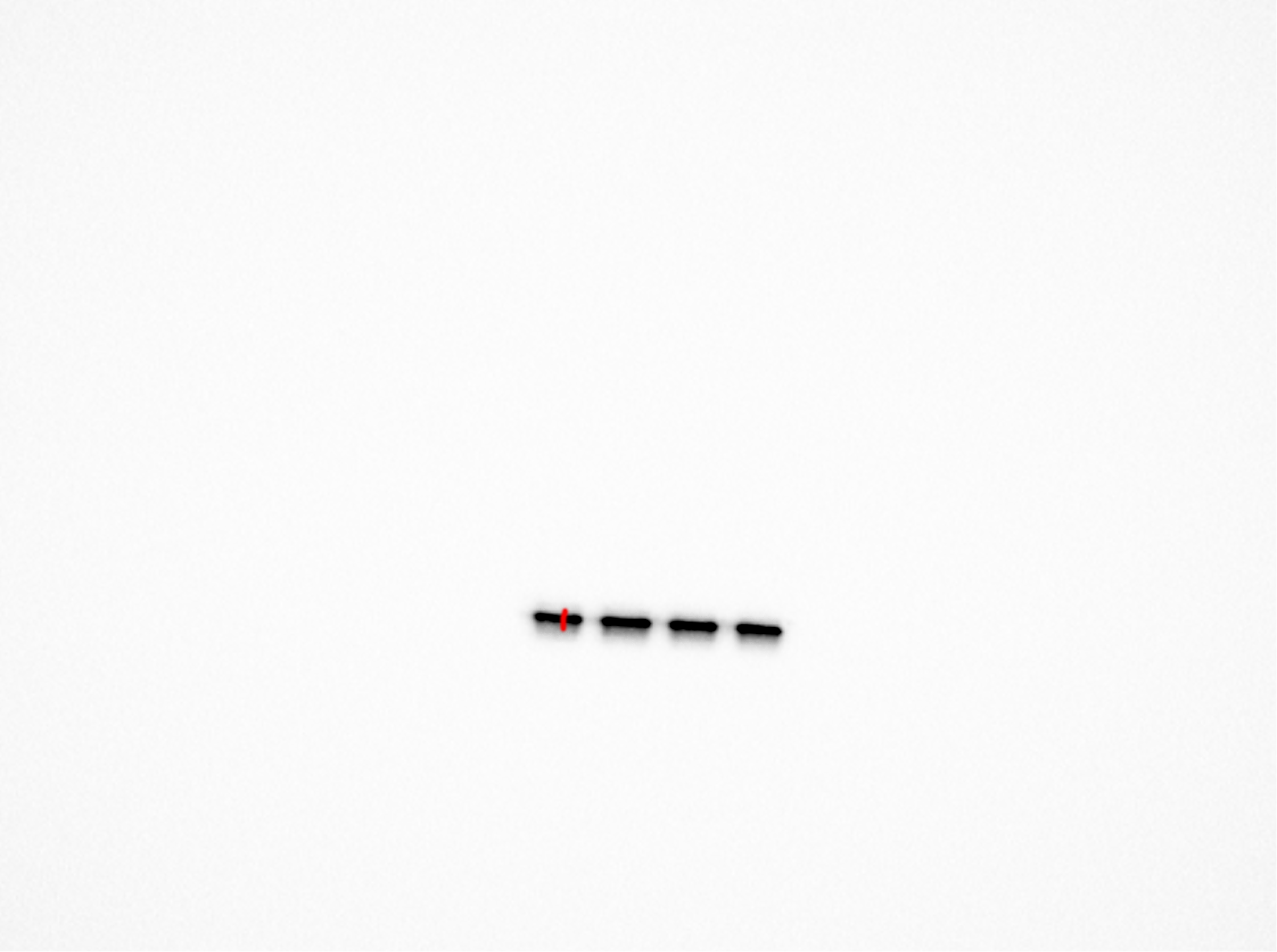

Supplement: Supplementary file 2 — full and uncropped western blots [file 41420_2022_941_MOESM2_ESM.docx]
